# Stability Analysis of the Fluorescent Tracer 1-Methylnaphthalene for IC Engine Applications by Supercontinuum Laser Absorption Spectroscopy

**DOI:** 10.3390/s20102871

**Published:** 2020-05-19

**Authors:** Peter Fendt, Ulrich Retzer, Hannah Ulrich, Stefan Will, Lars Zigan

**Affiliations:** Lehrstuhl für Technische Thermodynamik (LTT) and Erlangen Graduate School in Advanced Optical Technologies (SAOT), Friedrich-Alexander-Universität Erlangen-Nürnberg (FAU), D-91058 Erlangen, Germany; peter.fendt@fau.de (P.F.); ulrich.retzer@fau.de (U.R.); hannah.ulrich@fau.de (H.U.); stefan.will@fau.de (S.W.)

**Keywords:** absorption spectroscopy, laser-induced fluorescence tracer, LIF, 1-methylnaphthalene, fiber optic sensors, combustion diagnostics

## Abstract

1-methylnaphthalene (1-MN) is a widely used laser-induced fluorescence (LIF) tracer for planar imaging of mixture formation and temperature distributions in internal combustion (IC) engines. As the LIF measurement results can be biased by partial tracer oxidation, the conversion of 1-MN and the base fuel isooctane is analyzed in a calibration cell. First, measurements using supercontinuum laser absorption spectroscopy (SCLAS) are presented in order to quantify the conversion by detection of the produced H_2_O mole fraction. A single mode fiber (SMF) coupled setup is presented, with the fiber core acting as entrance slit of a Czerny-Turner spectrometer. Dependencies on residence time and global air-fuel ratio are presented at pressures up to 1.5 MPa and temperatures up to 900 K, at which distinct tracer and fuel consumption is observed. Signal loss due to intense beam steering was partially compensated using a self-stabilizing double-pass setup with a retroreflector.

## 1. Introduction

Laser-induced fluorescence (LIF) based on tracers is a well-established diagnostic technique for measurements in the gas, liquid, and solid phase. Especially for combustion applications, e.g., in IC engines or fuel sprays, the determination of the mixture composition or temperature [1,2,3,4] and droplet size [5,6] is of special interest. The tracer is usually added to a fuel, so that the thermo-physical and chemical properties of the tracer and the fuel should closely match to avoid a bias in the mixture formation, ignition, and combustion processes. Possible tracer decomposition, i.e., that the tracer may start to react, e.g., during compression in an IC engine while the fuel is not yet consumed, is a major problem that might be encountered in these measurements [1,7]. In principle, the auto-ignition temperature gives a rough indication of the chemical stability of fuel and tracer. However, the respective ignition time delay must also be considered. Usually, the residence time of the fuel and tracer in the hot ambience are relatively short during compression (few milliseconds), but relatively long for calibration measurements in a static chamber or flow cell (e.g., several seconds) [8]. Consequently, the LIF-calibration data may be biased in the case of pyrolysis and oxidation of the tracer at long residence times. Measurements of tracer (e.g., acetone) consumption utilized different analytic methods ranging from pressure increase to concentration measurements with gas chromatography, see, e.g., [9]. Kinetic studies in a shock tube exist for some tracers, but the conditions studied usually deviate from those present in IC engines and calibration cells [7]. Thus, in situ measurements during calibration are favorable to identify tracer consumption. 

Products of pyrolysis and secondary reactions may be detected as proof of tracer consumption. LIF spectra recorded in inert atmosphere (N_2_, CO_2_) in a calibration cell by Faust [10] were assigned to certain radicals or secondary reaction products of toluene and naphthalene. The stability of acetone was investigated under IC engine conditions in a flow cell while using Raman spectroscopy by Eichmann et al. [11]. There, the detection of the water signal was considered as a proof of tracer reaction. The partial tracer reaction occurred without a rise in temperature or pressure in the cell. Furthermore, with this technique, the reactions of toluene and 3-pentanone were studied in a calibration cell up to 3 MPa and 773 K for varied residence times of 1 s to 5 s [8]. Higher temperatures are usually studied in shock tubes [7,12], allowing for the direct measurement of ignition delays of fuels and fuel-tracer mixtures as well as comparison with kinetic simulations [12].

The tracer 1-MN is suitable for mixing studies at high temperate and pressure relevant for diesel IC engines. The uncertainty of this technique is in the range of 4.5% for the concentration and 3.2% for temperature measurements under calibration conditions [4]. However, the concentration measurements can be biased by tracer decomposition and oxidation. For 1-MN or mixtures of 1-MN with isooctane, no information is available on its chemical stability during LIF calibration under IC engine conditions [13,14]. In the present paper, we investigate the fuel/tracer reaction in a continuously scavenged calibration flow cell (HTC^2^) at various conditions and residence times via the detection of water formation while using near infrared (NIR) supercontinuum laser absorption spectroscopy (SCLAS). During calibration of the mixture, the tracer is dissolved in the surrogate fuel isooctane as it shows a relatively high auto-ignition temperature (as compared to many other diesel surrogate fuels). In general, the auto-ignition temperature only gives a rough indication of stability. For example, at standard pressure 1-MN shows an auto-ignition temperature of 530 K to 566 K, while isooctane is ignitable in the range of 415 K to 561 K, where these numbers reflect the ranges that are provided by the collection of Glassman and Yetter [15] and may vary due to the different test cells and materials used. Usually, also no information about tracer stability at increased pressure nor varied composition is available. Thus, for the evaluation of LIF-calibration data details about the chemical stability are necessary, especially for very high temperature and high pressure conditions in order to separate the effects of non-radiative processes (like fluorescence quenching etc.), temperature-dependent absorption cross section variation and tracer consumption on the measured LIF signal [13].

Laser absorption spectroscopy is a well-established non-intrusive method for measuring mole fractions of different species (*X*_i_), temperature (*T*), and pressure (*p*) within many applications [16,17,18]. Even under harsh conditions in combustion applications, e.g., in IC engines or Rapid Compression Machines (RCM), with rapidly changing temperature and pressure, various strategies exist for (multi-) parameter determination based on water absorption [18,19,20,21,22,23]. For the presented H_2_O mole fraction (*X*_H2O_) measurements in the calibration cell, a broadband SCLAS high-speed NIR spectrometer is used and data analysis is performed by a Voigt line-shape absorption model that is based on the HITEMP database [24,25,26]. For high pressure-high temperature conditions, using SCLAS is favorable when compared to comparatively narrow band tunable diode laser absorption spectroscopy (TDLAS), as broadband spectra provide information of line broadening effects and temperature dependent line strengths for several thousand absorption lines, thus improving the accuracy and robustness of the measurement [21,24,27]. In contrast to previous combustion measurements, where the transmitted beam was directly focused on the entrance slit of the spectrometer, the transmission signal is guided to the spectrometer by a SMF, with the fiber core acting as an entrance slit [22]. This approach is advantageous in several aspects. In general, a fiber-coupled sensor can be placed independently of the measurement section or line of sight (LOS). This is often advantageous in the case of limited space and/or harsh environmental conditions, e.g., for IC engine or gas turbine applications. Fiber coupling also allows for reducing the open optical path length. Thus, measurement errors due to a systematic bias (e.g., by water absorption in ambient gas. outside the region of interest) and signal fluctuations that are caused by variations of the refractive index (e.g., due to convection) can be reduced. In addition, the small size of the SMF core (e.g., 8.2 µm diameter) approximates an ideal point light source, which allows for a high spectral resolution. A SMF was chosen over a multi-mode fiber (MMF) approach, because of the modal noise and speckles induced in a MMF. If the MMF fiber core is covered by a spectrometer slit (e.g., 10 µm), only a fraction of the speckles can pass through the slit, resulting in intense broadband spectral features of the background signal. As a drawback, the small size of the SMF core complicates efficient coupling and alignment. In many applications, relative mechanical motions of optical components reduce coupling efficiency. In addition, beam steering (the alternation of the light path due to refractive index gradients) reduces the coupling efficiency and it leads to signal level fluctuations [28]. However, there are few scientific data using SMF-coupled SCLAS under intense beam steering conditions. In accordance with Locurto et al. [29], coupling the light back into the SMF fiber core is difficult, even within a moderate environment, such as a co-flow stabilized flat flame burner. There, the authors averaged 40,000 wavelength sweeps to obtain sufficient signal levels despite beam steering. A self-stabilizing retro reflector approach is tested in order to compensate for stronger and more dynamic beam steering present within the flow cell and to extend the optical path length. Retroreflective setups have been successfully applied in combustion applications, where the transmitted beam was either coupled to a MMF or directly focused on a detector [28,30,31]. Furthermore, significantly lower water mole fractions can be measured with shorter integration times when compared to the previously applied Raman technique. With the previous Raman and calibration cell setup, the minimum detectable water mole fractions were in the range of 2% to 2.8% (at 3 MPa, 698 K to 748 K, 60 s exposure time). Thus, starting partial decomposition of small fuel/tracer fractions is resolvable.

## 2. Materials and Methods

**Figure 1:**
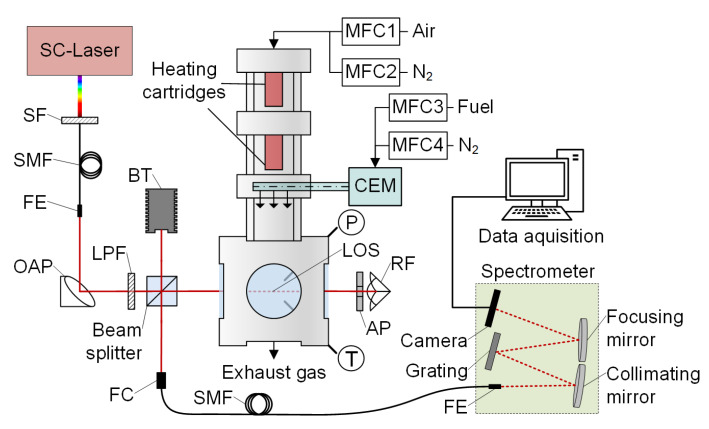
HTC^2^ calibration flow cell and the optical supercontinuum laser absorption spectroscopy (SCLAS) configuration. Optical components: spectral filter (SF), single mode fiber (SMF), fiber end (FE), off-axis parabolic mirror (OAP), longpass filter (LPF), beam trap (BT), aperture (AP), retroreflector (RF), and fiber collimator (FC).

Figure 1 shows the HTC^2^ with the fiber coupled SCLAS setup for H_2_O detection. Within the cell, temperature and pressure can be controlled in ranges of 300 K to 950 K and 0.4 MPa to 3 MPa, respectively.

The cell is insulated with ceramics, and temperatures are monitored with thermocouples for the vaporized fuel flow, below the heating cartridges and close to the LOS. Variable air-fuel ratios *AFR* and residence times *τ*_res_ are achieved by mass flow controllers (MFC, Bronkhorst, Ruurlo, The Netherlands), mainly adjusted by the main gas flow (variable mixture of nitrogen and dehumidified compressed air) injected from above. For temperature control, the flow is heated with two heating cartridges before it is merged with the fuel-nitrogen gas flow in the mixing section of the cell. Evaporation is controlled via fuel/tracer partial pressure in a controlled evaporator and mixing unit (CEM, Bronkhorst, Ruurlo, The Netherlands). The fuel-nitrogen mixture is then transferred to the mixing section at a constant temperature of 473 K. The fuel-gas flow is injected by six nozzles into the main gas flow, followed by a static swirl body. Local inhomogeneities in terms of temperature and air-fuel ratio might occur within the cell at short mixing times, which could bias the SCLAS-measurements. Planar LIF imaging was used in order to assess the mixing quality in the cell [32,33]. In an area of 10 mm by 10 mm in the center of the LOS, averaged spatial variations in concentration of 1% (standard deviation) and in temperature of 3.2% at 898 K (1.5 MPa) were determined. The latter was also confirmed by thermocouple measurements, with a maximum temperature gradient of 3.5% in a cell temperature interval from 600 K to 850 K along a 14 mm central section of the LOS path.

We calculate the residence time *τ*_res_ from the known total volume flow rate and the distance between the mixing zone and the LOS position. Flow rates are different for varying pressures in order to enable the respective temperature levels in the cell. 

The optical setup comprises a SC laser (NKT Photonics EXR-15, Birkerød, Denmark), a double pass LOS configuration, and a Czerny Turner spectrometer. The NIR part of the SC laser light is guided through a SMF (NKT Photonics) and collimated by an off-axis parabolic mirror (OAP, RFL = 50.8 mm). A beam splitter cube (50/50) and a gold-coated corner cube retroreflector double the LOS to a total path length of 164 mm inside the flow cell. An aperture (3 mm diameter) in front of the RF ensures a well-defined LOS position and it is part of the beam steering compensation: despite beam steering due to strong heat haze occurring within the flow cell, a significant fraction of the expanded and collimated light hits the center part of the corner cube RF. There, the lowest beam offset is generated and the light almost travels the same path when it passes the calibration cell for the second time. The beam is then partially reflected by the beam splitter to the collimator (OAP reflective fiber collimator) of the SMF. A standard SMF patch cable (SMF-28^®^ Ultra, NA = 0.14, 8.2 µm core diameter) with an increased length of 10 m is used in order guarantee sufficient attenuation within the fiber cladding, so that the size of the fiber core defines the spectrometer slit width. A NIR line camera (Sensors Unlimited GL2048L, Princeton, NJ, USA) is used to detect the spectrum within a range of 1328 nm to 1485 nm and a spectral resolution of 0.18 nm [24]. Flushing of the ambient optical path with dehumidified air (3.6% < relative humidity < 3.8%, monitored) ensures a constant low background absorption signal.

**Figure 2:**
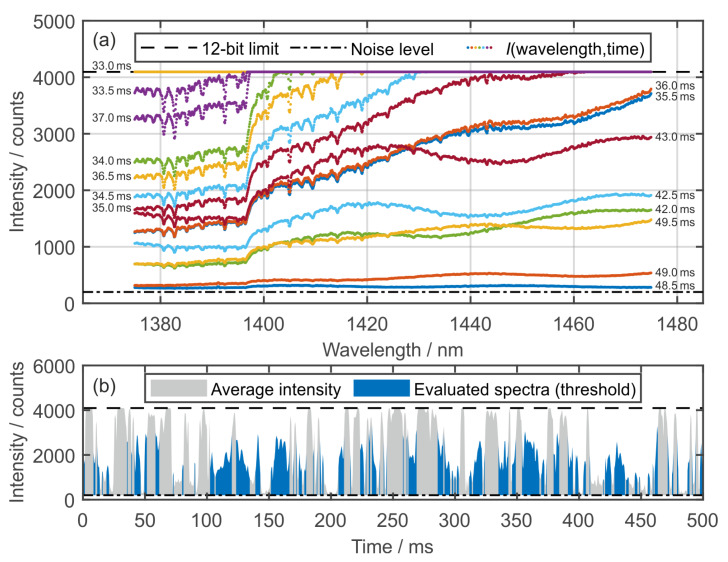
Examples of detected spectrometer signals (1 vol. % 1-MN in isooctane, 1 MPa 900 K, *τ*_res_ = 315 ms, *X*_H2O_ = 0.98%). (**a**) Typical transmission spectra *I* (colors represent single frames at manual selected time steps, smoothed) with temporal variations in signal intensity; and, (**b**) temporal course of spectral averaged signal intensity and fraction of evaluated (threshold filtered) spectra.

For the measurements, the calibration cell is first set to the desired pressure and temperature level at deactivated fuel/tracer flow until a stationary condition is reached. Subsequently, the measurement of the background signal *I*_0_ is carried out. The fuel/tracer flow is subsequently switched on, followed by the measurement of the signal *I* with the presence of a steady mass flow rate. 5000 consecutive frames each are captured for background and absorption measurements, respectively, at a 2 kHz detection rate, resulting in 2.5 s overall measurement time. In Figure 2, the typical transmission spectra detected by the spectrometer and the consecutive average camera intensity for a measurement section of 1000 frames are shown. 

Over- and poorly-exposed frames are sorted out through automated threshold filtering. The filter removes all of the images where either the 12-bit camera limit (4095 counts) or a minimum exposure intensity of 400 counts (without dark noise) is violated more than 10 times. While the average intensity fluctuates significantly due to beam steering, the spectral distribution of the background intensity (*I*_0_) remains quite stable (see Figure 2a). Broadband fluctuations, probably caused by beam steering and interference fringes, as shown in the overlapping spectra, can be automatically compensated for during data evaluation, for example, by differentiation [27], Bayesian inference and fringe modeling [34], or a Fast Fourier Transformation [35]. For most of the measurements, a larger fraction of overexposed frames is observed, as visualized in the temporal course of the average camera intensity (Figure 2b). This effect is increased by the transmission properties of the optical components used, resulting in an increasing spectral intensity above 1395 nm. The applied filtering results in approximately 1000 to 2000 frames that are averaged for each presented measurement within this work, thereby significantly improving the signal to noise ratio and, thus, expanding the absorption detection limit. With changes to the optical components, we estimate the part of threshold-filtered poor quality frames to be below 30% based on the low intensity data below 1395 nm for the given operating point. In general, the sorted out part of frames is very sensitive to the flow conditions within the cell and reached a minimum of about 10% at higher flow rates (700 K to 900 K, 5 MPa, *τ*_res_ = 105 ms). Further improvements could be achieved by a dynamic adaption of the evaluated spectral range.

With regard to the data evaluation, only a brief overview with major changes in the evaluation is provided, and a detailed description of the underlying model is given in [24,25]. The absorbance (*A*) spectra are calculated from the averaged transmission signals *I* and *I*_0_ according to Lambert–Beer’s law. A nonlinear least squares (NLLSQ) curve fitting algorithm based on the MATLAB^®^ “lsqnonlin”-solver iteratively optimizes a modeled H_2_O Voigt line-shape absorbance (integrated) spectrum for a best fit with the measured absorbance (Equation (1)):

(1) $$A=-\ln\left(\frac{I}{I_{0}}\right)=\sum S_{i}\left(T\right)\phi_{i}\left(\lambda ,p,T,X_{H2O}\right)pX_{H2O}L.$$

**Figure 3:**
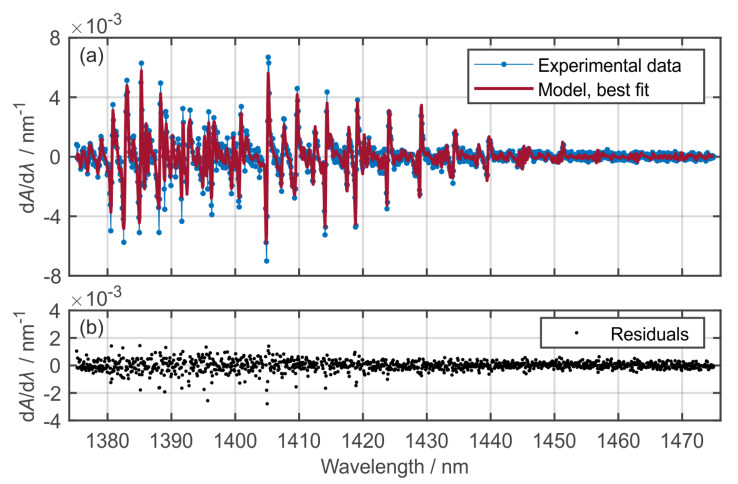
Example for spectra evaluation using NLLSQ curve fitting. (**a**) Best fit for an example dataset (1 vol. % 1-MN mixture consumption resulting in *X*_H2O_ = 0.25% at *p* = 1.5 MPa, *T* = 850 K); (**b**) residuals.

In this model, *S*_i_ is the temperature (*T*) dependent line strength, *ϕ* is the Voigt line shape function, depending on wavelength *λ*, pressure *p*, temperature, and mole fraction, and *L* is the LOS path length. The optimization is based on the derivative d*A*/d*λ* in order to reduce the impact of broadband spectral deviations between *I* and *I*_0_ (Figure 3) [27].

The measured pressure from the pressure transducer is used as input parameter and solely temperature and H_2_O mole fraction remain as optimization variables. Hence, the *X*_H2O_ uncertainty and precision are improved as the pressure and mole fraction are significantly correlated within this broadband fitting approach [24]. Temperature inhomogeneities are approximated with a simple two-zone temperature model to account for observed lower temperatures due to the heat loss close to the windows of the calibration cell. Such models are often used to estimate temperature non-uniformities [22,36] or the occurring maximum temperature [37]. Within the model, the LOS is separated into a core zone with higher temperature and a combined window zone with lower temperature, both separately modeled and combined within the routine. However, when compared to the assumption of a homogeneous temperature distribution, the relative difference in H_2_O mole fraction is less than 3% for the presented parameter range. This confirms that the unknown exact temperature distribution only has a minor effect on the *X*_H2O_ evaluation. 

A previously conducted study with vaporized water was used to estimate the SCLAS mole fraction uncertainty. According to Lind et al. [38], we estimate the predefined mole fraction at *X*_H2O_ = 1% ± 0.04% by error propagation based on the uncertainties of the flow cell for adjusting the water steam flow (3.2%) and air flow (2.5%). In an interval between 700 K and 900 K for pressures between 1 MPa and 1.5 MPa, respectively, the NLLSQ absorbance algorithm evaluated *X*_H2O_ = 0.96% ± 0.03% based on eight samples. A minimum H_2_O mole fraction of approx. 120 ppm could be reproducibly detected for pure 1-MN at 850 K and *AFR* = 0.5.

## 3. Results and Discussion

This study investigated a mixture of 1 vol. % 1-MN in isooctane and the base components 1-MN and isooctane. The air is highly diluted with nitrogen according to Table 1 due to safety issues, thereby inhibiting the chemical reaction.

**Figure 4:**
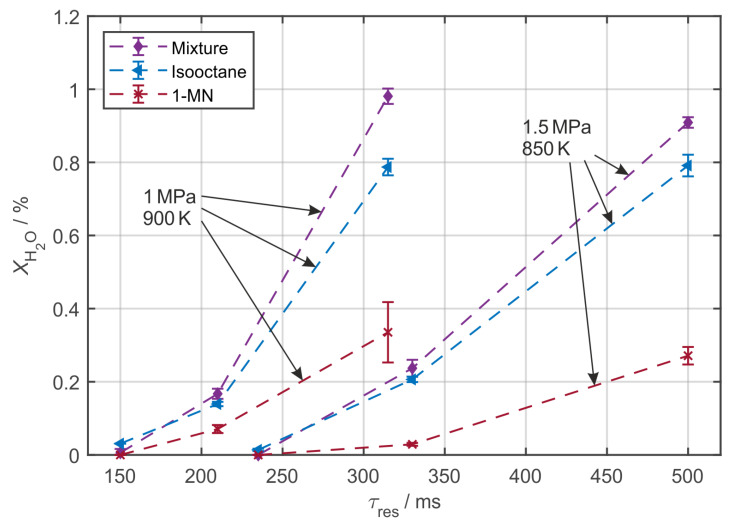
Residence time variation for 1 vol. % 1-MN in isooctane, pure isooctane and 1-MN for two operating points (*AFR* = 1).

Data are shown for two operating points with deviating temperature and pressure, at which distinct residence time and *AFR* dependencies of the H_2_O mole fraction are observed. In Figure 4, the measured residence time dependencies of *X*_H2O_, which correlate with fuel/tracer oxidation, are shown at *AFR* = 1.

In general, the error bars represent the sample standard deviation (SD) of at least three independent measurements. The *X*_N2_/*X*_O2_-ratios for *τ*_res_ = 210 ms and *τ*_res_ = 330 ms (*AFR* = 1) are specified according to Table 1 and six independent measurements were performed and evaluated for the SD at both of these residence times. The mole fractions for measurements without any recognizable H_2_O absorption are manually set to zero. Assuming a full consumption of the fuel/tracer mixture and a simple global reaction equation, a theoretical upper limit of *X*_H2O,max_ = 0.95% is expected for pure isooctane (*X*_H2O,max_ = 0.43% for pure 1-MN). From the two operating conditions, a significant temperature dependency is observed: At 900 K and 1 MPa, the mixture is fully consumed at *τ*_res_ = 315 ms, while oxidation just started for the 1.5 MPa 850 K operating point. For 850 K cell temperature, lower oxidation rates are observed for 1-MN at long residence times, from which a higher auto ignition temperature may be derived. Full conversion residence times could not be achieved for all cases as the residence time interval is limited due to the available MFCs. For the mixture of 1-MN in isooctane, higher average H_2_O mole fractions are measured when compared to pure isooctane for both operation points. The fuel/tracer mixture seems to be more reactive than either base component. Sommerer et al. [12], who studied 10 vol. % tracer mixtures in isooctane, found a preceding consumption of the aromatic tracers methylbenzene (toluene) and trimethylbenzene at low temperature (850 K) fuel oxidation conditions. However, the authors provided no information on the conversion rate for the fuel/tracer mixture when compared to the base components.

**Figure 5:**
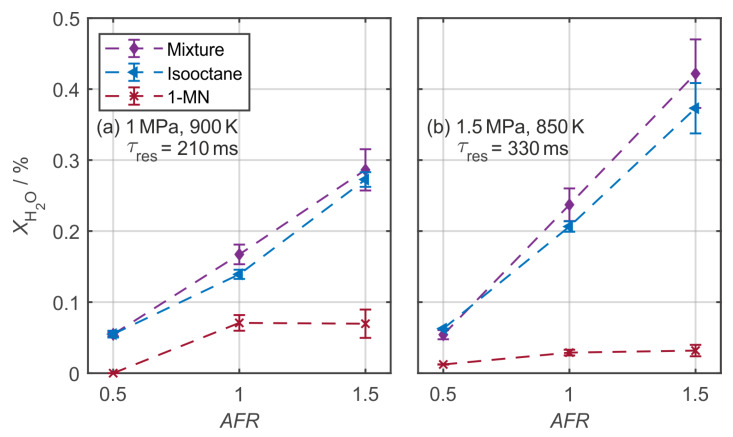
Air-fuel ratio dependency for pure isooctane, 1-MN and 1 vol. % 1-MN in isooctane, for two operating points (**a**,**b**).

Figure 5 shows the air-fuel ratio dependent fuel/tracer conversion for the two operating conditions. 

Pure isooctane and the 1 vol. % 1-MN/isooctane mixture both exhibit an almost linear relation between air-fuel ratio and water production. We attribute this dependency to an increasing oxygen concentration in the mixture as the *AFR* is solely controlled by the air-nitrogen composition of the main gas flow. For higher *AFR*s a lower nitrogen dilution is performed, leading to a higher oxygen concentration. This increases the frequency for collisions of the reactants, thus increasing reactivity and conversion. For both residence times, based on the averaged values, the 1 vol. % 1-MN mixture shows a higher reactivity when compared to pure isooctane. Pure 1-MN seems to deviate from a linear behavior, and, considering the lower theoretical upper limit of *X*_H2O,max_ = 0.43%, a significantly lower conversion at a temperature of 850 K (Figure 5b) is observed. This is in accordance with the higher ignition temperature (or lower reactivity when compared to isooctane) of pure 1-MN. 

## 4. Conclusions

In conclusion, a first study on the chemical stability of 1-MN/isooctane mixtures was conducted under IC engine relevant conditions while using SCLAS. The residence time and air-fuel ratio dependencies highlight similar dependencies between the 1 vol. % 1-MN/isooctane mixture, pure isooctane and 1-MN: a slightly increased reactivity of the mixture compared to pure isooctane is observed, whereas pure 1-MN shows the lowest reactivity, especially for the lower cell temperature of 850 K. With sensitivity in the order of 100 ppm H_2_O mole fraction, the double pass SCLAS setup is capable of resolving a partial consumption of the fuel/tracer mixtures. Furthermore, despite significant beam steering within the calibration cell, the use of a single mode fiber core as an entrance slit of the spectrometer has proven to be highly suitable within this study. Future efforts will be directed at using the SCLAS approach with an SMF-coupled spectrometer for multipass measurements in a fired RCM.

## Figures and Tables

**Table 1:** Operating conditions of the flow cell.

Parameter	Unit	Substance/Operating Points		
		1-MN	Isooctane	Mixture
*X*_N2_/*X*_O2_		85	75	75
*X*_O2_/*X*_fuel/tracer_		13.5	12.5	12.5
*X*_H2O,max_	%	0.43	0.95	0.94
		1 MPa, 900 K	1.5 MPa, 850 K	
*τ*_res_	ms	150, 210, 315	235, 330, 500	
*τ*_res_ (*AFR*)	ms	210	330	
